# Perceptual Accent Rating and Attribution in Psychogenic FAS: Some Further Evidence Challenging Whitaker’s Operational Definition

**DOI:** 10.3389/fnhum.2016.00062

**Published:** 2016-03-02

**Authors:** Stefanie Keulen, Jo Verhoeven, Roelien Bastiaanse, Peter Mariën, Roel Jonkers, Nicolas Mavroudakis, Philippe Paquier

**Affiliations:** ^1^Clinical and Experimental Neurolinguistics (CLIEN), Vrije Universiteit Brussel, Brussels, Belgium; ^2^Center for Language and Cognition Groningen (CLCG), Rijksuniversiteit Groningen, Groningen, Netherlands; ^3^Computational Linguistics and Psycholinguistics Research Center (CLIPS), Universiteit Antwerpen, Antwerp, Belgium; ^4^Department of Language and Communication Science, City University London, London, UK; ^5^Department of Neurology and Memory Clinic, ZNA Middelheim General Hospital, Antwerp, Belgium; ^6^Department of Neurology, Erasme University Hospital, Université Libre de Bruxelles, Brussels, Belgium; ^7^Unit of Translational Neurosciences, Universiteit Antwerpen, Antwerp, Belgium

**Keywords:** foreign accent syndrome, psychogenic, speech disorder, agrammatism, perceptual experiment, bi- and multilingualism

## Abstract

A 40-year-old, non-aphasic, right-handed, and polyglot (L1: French, L2: Dutch, and L3: English) woman with a 12-year history of addiction to opiates and psychoactive substances, and clear psychiatric problems, presented with a foreign accent of sudden onset in L1. Speech evolved toward a mostly fluent output, despite a stutter-like behavior and a marked grammatical output disorder. The psychogenic etiology of the accent foreignness was construed based on the patient’s complex medical history and psychodiagnostic, neuropsychological, and neurolinguistic assessments. The presence of a foreign accent was affirmed by a perceptual accent rating and attribution experiment. It is argued that this patient provides additional evidence demonstrating the outdatedness of Whitaker’s (1982) definition of foreign accent syndrome, as only one of the four operational criteria was unequivocally applicable to our patient: her accent foreignness was not only recognized by her relatives and the medical staff but also by a group of native French-speaking laymen. However, our patient defied the three remaining criteria, as central nervous system damage could not conclusively be demonstrated, psychodiagnostic assessment raised the hypothesis of a conversion disorder, and the patient was a polyglot whose newly gained accent was associated with a range of foreign languages, which exceeded the ones she spoke.

## Introduction

Foreign accent syndrome (FAS) is a speech-output disorder, which affects the segmental and suprasegmental characteristics of speech in such a way that a speaker is no longer able to make the correct phonetic/phonematic contrasts of his/her native language. The FAS speaker is interpreted by listeners to be a non-native speaker of his/her mother tongue, or – in some cases – as speaking a different dialectal variant. Numerous cases of FAS have been attested since Marie (1907) described the case of a Parisian man who started speaking with an Alsatian accent after having sustained an intracerebral hemorrhage, which Marie localized at the level of the left lentiform nucleus. Reeves and Norton (2001) were the first to explicitly link their schizophrenic patient’s foreign accent (syndrome) to his psychotic exacerbations. Before them, Critchley (1962) and Gurd et al. (2001) had already hinted at a possible psychogenic etiology for FAS their patients had developed. However, they did not label it as such, possibly due to a lack of objective proof, and because in the context of Whitaker’s (1982) operational definition (Table 1), the possibility of a psychogenic FAS is excluded. According to Whitaker’s criteria, indeed, FAS is strictly related to central nervous system damage.

**Table 1:** **Whitaker’s operational definition of FAS (Whitaker, 1982; pp. 196 and 198)**.

(1) The accent is considered by the patient, by acquaintances, and by the investigator, to sound foreign
(2) It is unlike the patient’s native dialect before cerebral insult
(3) It is clearly related to central nervous system damage (as opposed to an hysteric reaction, if such exists)
(4) And there is no evidence in the patient’s background of being a speaker of a foreign language (i.e., this is not like cases of polyglot aphasia)

In 2005, Van Borsel et al. defended the hypothesis of a psychogenic FAS in their 32-year-old female patient, who presented with FAS, as well as with subtle grammatical anomalies. Medical history revealed she had suffered from depression and suicidal ideation. A neurological and radiological work-up did not reveal any neurological deficit. Other psychogenic case studies would follow (Verhoeven et al., 2005; Poulin et al., 2007; Reeves et al., 2007; Cottingham and Boone, 2010; Haley et al., 2010; Jones et al., 2011; Lewis et al., 2012; Roy et al., 2012; Polak et al., 2013). Close inspection of the FAS case studies – irrespectively of etiological substrate – reveals that this disorder rarely occurs as a “stand-alone phenomenon.” Rather, there is a rich spectrum of possible comorbid speech and language impairments that can accompany FAS. The most common comorbid speech and language disorders of neurogenic FAS are dysarthria, apraxia of speech, and aphasia, mostly of the non-fluent type, although the fluent type has also been reported. In addition, muteness has been reported as a speech disorder frequently preceding FAS (Table 2). Furthermore, specific language impairment (SLI), developmental apraxia of speech (DAS), and agrammatism (mainly in the context of aphasia) have been noted.

**Table 2:** **Overview of the comorbid speech and language disorders in neurogenic FAS cases**.

Comorbid speech and language disorders	Reference
Dysarthria	e.g., Monrad-Krohn (1947) and Nielsen and McKeown (1961), cases 1 and 2; Whitty (1964), Schiff et al. (1983), Graff-Radford et al. (1986), and Berthier et al. (1991), cases 1 and 2; Scianna et al. (2000)
Apraxia of speech	e.g., Whitty (1964), Ingram et al. (1992), Takayama et al. (1993), and Laures-Gore et al. (2006), case 1
Aphasia: fluent	e.g., Kwon and Kim (2007) and Katz et al. (2008)
Aphasia: non-fluent	e.g., Monrad-Krohn (1947) and Nielsen and McKeown (1961), case 1; Graff-Radford et al. (1986), Ardila et al. (1988), and Berthier et al. (1991), cases 3 and 4
Mutism (pre-FAS)	e.g., Gurd et al. (1988) and Berthier et al. (1991), cases 1 and 2, Roth et al. (1997), Carbary et al. (2000), and Munson and Heilman (2005)

In psychogenic FAS, the only comorbid speech/language impairments that have been attested over the years are a “pre-FAS”-muteness (Van Borsel et al., 2005; Jones et al., 2011), and grammatical anomalies (Van Borsel et al., 2005; Verhoeven et al., 2005; Poulin et al., 2007; Cottingham and Boone, 2010). The previously mentioned 32-year-old right-handed patient reported by Van Borsel et al. (2005) presented with grammatical errors, not explicable by any neurological damage. Aberrant realizations concerned substitution errors (mainly affecting nouns and verbs), omissions (especially affecting auxiliaries, prepositions and articles), and a dyssyntaxis. Importantly, the authors note that this pattern of grammatical errors did not conform to the pattern typically seen in Broca (agrammatism) or Wernicke (paragrammatism) aphasia. FAS in their patient found its expression through segmental alterations (e.g., “devoicing of voiced consonants,” “cluster reduction of r-clusters,” “initial consonant deletion of /h/”; p. 423) and suprasegmental alterations (“improper word stress,” “improper sentence stress,” “a tendency toward scanning speech”; p. 423). Two years later, Poulin et al. (2007) (Roy et al., 2012) diagnosed FAS in a 74-year-old, bipolar patient. Although they doubted the psychogenic origin of FAS, consensus as to the psychogenicity in their patient was subsequently reached among other authors (Mariën et al., 2009; Haley et al., 2010; Jones et al., 2011; Lewis et al., 2012). Other instances of psychogenic FAS in bipolar patients would follow (Reeves et al., 2007; case 2). Poulin et al.’s (2007) patient demonstrated “mild agrammatism.” In contrast to what is generally seen in Broca aphasia patients with a marked agrammatism, the speech of their patient was fluently^1^ produced, though it was telegraphic in structure. Function words as well as bound grammatical morphemes were omitted. Unfortunately, the possible occurrence of a (multimodal) grammatical disorder in written language production was not investigated. In 2010, Cottingham and Boone described a 36-year-old woman implicated in a motor vehicle accident (MVA), who developed an Eastern European-like accent 3 years after the MVA occurred. She too developed a “telegraphic style” of speech. A combination of different arguments pleaded for the psychogenic etiology of her accent shift. First, there was the late onset of the accent (3 years post-MVA). In addition, there were no demonstrable anomalies on MRI and EEG. Furthermore, the patient exhibited a left-sided give-way weakness. Linguistically, she demonstrated difficulties in sentence repetition (10 days post-MVA), which were limited to the clinical test setting, as well as improbable error patterns (splitting numbers into separate digits).^2^ There were irregularities in the (aberrant) intonation pattern and inconsistencies in the grammatical disorder (deleting a preposition in one sentence, using it in the following sentence and then deleting it again). Lastly, her answers on the Minnesota Multiphasic Personality Inventory (MMPI-2) (Butcher et al., 1989), although less conclusively than expected (possibly influenced by a defensive stance), indicated a hysterical personality orientation (suggesting conversion disorder).

The current paper adds to the literature on psychogenic FAS by presenting a new case challenging Whitaker’s (1982) operational definition. We report a 40-year-old, non-aphasic woman, with a 12-year history of addiction to psychoactive substances, who presented at the Erasme University Hospital neurology department between 2010 and 2013, with a complex and diverse set of symptoms mainly perturbing her gait and language. Alterations affecting her oral verbal output were stutter-like behavior, (atypical) grammatical errors,^3^ as well as FAS. Based on an analysis of her complex medical history, the psychodiagnostic neuropsychological, and neurolinguistic assessments, we advance the hypothesis of a psychogenic etiology. This case report also demonstrates that identifying the provenance of the perceived accent foreignness depends on the listener’s subjective impression.

## Background

In a study on the pathophysiological mechanism of different speech disorders, Whitaker (1982) assigned four characteristics to the speech disorder, which he coined “foreign accent syndrome” (Table 1). As is clear from our introduction, many case reports have defied one or more criteria proposed by Whitaker. This has been an incentive for the conceptualization of a distinctive taxonomic variant of FAS, *psychogenic FAS*, which is instigated by psychological or psychiatric problems (Verhoeven and Mariën, 2010).

The aim of the current study is twofold: (1) based on the medical history, the symptoms at presentation, the neurocognitive work-up, as well as the psychodiagnostic and neurolinguistic assessments, we argue that the nature and evolution of the patient’s speech/language symptoms are highly indicative of a psychogenic etiology and (2) we experimentally corroborate the hypothesis of FAS by performing an accent rating and attribution task (Verhoeven et al., 2013).

### Patient and Medical History

The patient gave informed written consent to report her data according to the standards and regulations established by the ethics committee of the Erasme Hospital (ULB).^4^

The patient is a 40-year-old, right-handed, Belgian woman with 13 years of education. She is an unbalanced polyglot speaker: she was raised in French (L1) and Dutch (L2) as an early bilingual, and learned English (L3) at secondary school. French is her everyday language. She sustained a cerebral concussion after a fall at age 17. She had suffered from severe addiction to multiple opiates and psychoactive substances (cocaine, LSD, cannabis, etc.) for a period of 12 years (1988–2000). In 2003, she benefited from a last inpatient withdrawal treatment, after which she self-admittedly stated to have been clean. In 2005, she was admitted to the same psychiatric institution because of somatization, insomnia, anxiety, underfeeding, and abulia, probably resulting from an anxio-depressive decompensation. She was considered to exhibit a histrionic personality disorder. An EEG was normal. She was discharged after a month of intensive psychotherapy, and remained under antidepressant and anxiolytic medication. She underwent surgery for a C5–C6 cervical hernia in 2008.

In February 2010, the patient was readmitted to the psychiatric institution because of speech problems of sudden onset,^5^ characterized by telegraphic speech, stuttering, and a change of accent especially when speaking French. She also complained of attention problems, nuchal pain, and arthralgia. She presented with non-rhythmic myoclonic jerks in lower limbs disturbing her gait, but clinical neurological examination revealed no motor or sensory deficits. Tendon reflexes were normal, and there was no cerebellar dysmetria. CT scan and MRI of the brain were normal, as was an EEG. Clinical biology tests revealed no abnormalities. Bone scintigraphy, cervical CT scan, and echography of uterus were all normal. Incidentally, at one occasion, she was noticed to speak normally during a temper tantrum caused by a feeling of not being taken seriously by the nursing staff.

In June 2010, the patient was seen at the neurological outpatient clinic for complaints concerning gait and speech. The gait and language abnormalities could not be explained by any neurologically induced deficits, and the hypothesis of a conversion disorder as well as Münchausen syndrome was formulated. In July 2010, the patient was hospitalized for largely the same complaints as the month before: (unstable) gait, backache, as well as impaired speech and language. The most striking speech symptoms consisted of a telegraphic output and stuttering (affecting her French), along with a change of accent (all formally attested during neurolinguistic investigation). Clinical neurological examination, CT scan of the brain, and clinical biology tests (HIV, mycoplasma, HCV, HBV, syphilis, and *Borrelia*) were completely normal. An MRI of the brain, performed prior to the current admission, was reported to be normal except for a discrete cortico-subcortical atrophy. An EEG was inconclusive because of the presence of muscular artifacts. Because of the multiple complaints of cervical and joint pain, a second follow-up was initiated at the outpatient algologic clinic. In September 2010, she was initially seen at the neurological outpatient clinic but was hospitalized because she repeatedly fell (admitted after a fall out of a wheelchair) and had diffuse pain complaints (especially situated near the cervical disks). Psychiatric complaints were noted after admission. The patient showed a behavioral regression limiting her autonomy.

In April 2011, the patient was again admitted for approximately 1 month to the psychiatric ward because of depression, insomnia, and regression of her physical state. When hospitalized, medical staff equally noted a behavioral regression to an infantile state: the patient was incontinent (wore diapers), had cuddly toys in her hospital bed, used a pacifier, and kept herself in fetal position. Clinical neurological examination, CT scan of the brain, and an EEG were all normal. In May 2011, she received a full neurolinguistic work-up (*see below*), which demonstrated deficits affecting all language faculties. The foreign accent, articulatory efforts, and stuttering had diminished compared to June 2010. The grammatical output disorder, which affected her (fluent) speech as well as writing, was still perceptible. The neurolinguist concluded that the speech and language symptomatology was unlikely caused by a neurological disorder. The follow-up notes of the algologist until February 2012 did not mention any improvement of speech and language.

In August 2012, she was seen at the neurological outpatient clinic. At that time, she was wheelchair-bound due to sudden immobility of the lower limbs and hypoesthesia of the left hemicorpus. A last neurolinguistic work-up was realized, which demonstrated that the grammatical disorder was still present in writing, but no longer in speech. The foreign accent also had disappeared, and stuttering had remarkably diminished compared to May 2011. Language problems had – according to the patient – spontaneously resolved after she woke up from an appendectomy under general anesthesia performed 1 month earlier in a peripheral hospital.

The last time the patient was seen at the neurological outpatient clinic in July 2013, oral language production was normal. The patient presented with a complex clinical picture associating a fibromyalgic syndrome, osteo-articulatory pain, arthrosis, and a cervical discopathy. Because of the spontaneous resolution of her speech and oral language problems, the patient no longer sought neurological advice at our institution.

### Psychodiagnostic Assessment

Psychodiagnostic assessment was conducted in 2010 by means of a structured interview, the Rorschach Test (Rorschach, 1921; Rorschach and Oberholzer, 1923), and the Object Relations Technique (Shaw, 2002). Results revealed passive self-reflection and infantile tendencies in thought, which had not (yet) found expression in her actions (this was the case in April 2011; see Patient and Medical History). The psychodiagnostic examination did not indicate a psychological dissociation. According to her Rorschach test results, the patient had regressed to an “archaic” stadium, which caused her to be nervous and which could have been incited by a fear to enter “the adult world,” possibly due to traumatic events she experienced as a child (tumultuous relationships with her parents and relatives). Based on the neurological and psychiatric examinations, and given the numerous somatic complaints for which no organic lesions could be demonstrated, the patient was considered to suffer most likely from a “hysterical conversion disorder,” although this was not substantiated by formal psychodiagnostic testing (she refused to be administered the MMPI).

### Neuropsychological Assessment

Standardized neuropsychological tests were carried out in 2010 (Table 3). The patient had an estimated premorbid IQ of 92 (Beauregard, 1971), which corresponded to an IQ of 91 as measured by the Raven Progressive Matrices (Raven et al., 1996). Verbal reasoning was normal according to the WAIS-Similarities subtest (Wechsler, 1970). The patient’s short-term memory was normal in the visuospatial modality as measured by the Corsi block-tapping test (Milner, 1971) and the Violon Beehive Test (Violon and Wijns, 1984) but slightly defective in the verbal modality according to the WAIS-Digit Span (Wechsler, 1970). Delayed memory was impaired both in the visuospatial modality as assessed by the Benton Visual Retention Test (Benton, 1953) and the Rey–Osterrieth complex figure test (ROCF) (Rey, 1941), and in the verbal modality in agreement with the Rey Auditory-Verbal Learning Test (RAVLT) (Rey, 1964). Free verbal recall was normal according to the Wechsler Memory Scale-Logical Memory (Wechsler, 1969), but the RAVLT (Rey, 1964) showed decreased verbal learning. Visuoconstructive skills were normal, and there were no signs of visual neglect on the ROCF (Rey, 1941). The patient showed normal performance on Part A of the Trail Making Test (Reitan, 1992; Godefroy, 2008), but Part B indicated decreased speed for attention and sequencing. This could not be confirmed by the WAIS coding subtest (Wechsler, 1970). Of note, during the neuropsychological assessment, the psychologist also discerned a “German/Slavic”-like accent, along with a severe grammatical anomaly in spontaneous speech.

**Table 3:** **Neuropsychological test results (September 2010)**.

Test	Raw score (/max. score) (st. = standard score)	Percentile	Mean	SD
**MEMORY**				
**Wechsler Memory Scale**				
Verbal span (direct/reverse)	7		10.54	1.92
Logical memory	9		10.43	3.07
Block-tapping test of Corsi	4		5.11	1.01
**Benton drawings**				
Immediate recall	3 (/10)			
Delayed recall	10 (/25)			
**Rey complex figure**				
Model	I	Pc. 50–100		
Time	2′	Pc. 100		
Score	14	Pc. <10		
The beehive test (Violon)				
Memory				
First trial	2		5.6	2.72
Second trial	8		7.8	2.07
Third trial	8		8.65	2.21
Fourth trial	10		9.1	2.05
Fifth trial	10		9.45	1.32
**15 words of Rey**				
Recall: total *n* words	42	Pc. <25		
First trial	8	Pc. 50		
Third trial	8	Pc. 0		
Fifth trial	6	Pc. 0		
**ESTIMATED PREMORBID IQ**				
Verbal automatisms of Beauregard	21 (/40)	Pc. 25 (IQ: 92)		
**INTELLECTUAL FUNCTIONS**				
Raven matrices	30 (/60) (IQ: 91) (time: 32′)			
WAIS – similarities	17 (/26) (st. = 10/20)			
**PRAXIS**				
Rey complex figure		
Model	I	Pc. 50–100		
Time	2′10″	Pc. 75		
Score	26	Pc. <10		
**CONCENTRATION, ATTENTION, AND MENTAL CONTROL**				
WAIS coding	35 (st. = 8/20)		33.55	1.4
**EXECUTIVE FUNCTIONS**				
Trail Making Test
Time (A)	35″	Pc. 50–75	31″	12
Error (A)	0	Pc. 5–75	0.12	0.45
Time (B)	135″	Pc. >95	66″	24
Error (B)	2	Pc. 5	0.14	0.46

*Pc., percentile*.

### Neurolinguistic Assessment

Neurolinguistic assessments took place in July 2010, May 2011, and August 2012 by means of a series of standardized tests (Table 4), and repeatedly failed to evidence aphasia.

**Table 4:** **Neurolinguistic test results**.

Test (/max. score)	Scores July 2010	Scores May 2011	Scores August 2012	Cut-off
**Oral comprehension**				
Word discrimination BDAE (/72)	70	68.5	71	67
Body-part identification BDAE (/20)	18	18	18	18
Commands BDAE (/15)	14	15	15	13
Token Test (/36)	27	27	31	29
**Oral expression**				
Non-verbal agility BDAE (/12)	6	NA	7	9
Verbal agility BDAE (/14)	6	6	11	11
Automatized sequences BDAE (/8)	8	6	NA	6
**Repetition BDAE**				
Words (/20)	15	18	20	18
High probability repetition (/8)	3	3	8	6
Low probability repetition (/8)	2	2	8	6
Responsive naming BDAE (/30)	29	30	30	27
Naming Bachy 36 items (/36)	27	31	29	35
Body-part naming BDAE (/30)	30	27	30	24
Verbal fluency (animals) (1 min)	18 (pc. 25)	15 (pc. <10)	11 (pc. <10)	16.5
(2 min)	26	22	17	24.5
**Reading**				
Word reading BDAE (/30)	27	24	30	30
Sentence reading BDAE (/10)	1	4	10	8
Symbol and word discrimination BDAE (/10)	NA	8	NA	10
Word-recognition BDAE (/8)	NA	8	NA	6
Comprehension of oral spelling BDAE (/8)	NA	8	NA	6
Word/picture matching BDAE (/10)	10	NA	NA	10
Reading sentences and paragraphs BDAE (/10)	9	8	6	7
**Writing**				
Writing mechanics BDAE (/5)	5	5	5	5
Serial writing BDAE (/47)	NA	46	NA	47
Primer-level dictation BDAE (/15)	NA	15	15	15
Spelling to dictation BDAE (/10)	8	8	7	6
Sentences to dictation BDAE (/12)	NA	NA	10	10
Written confrontation naming BDAE (/10)	8	10	NA	7
Narrative writing BDAE (/5)	3	3	3	4

*NA, not administered; Pc., percentile*.

#### Auditory Comprehension

Auditory comprehension was assessed using the French version of the Boston Diagnostic Aphasia Examination (BDAE) (Mazaux and Orgogozo, 1983) and the shortened Token Test (De Renzi and Faglioni, 1978). Except for the Token Test, results were well within the normal range on the three occasions. Both Token Tests administered (2010 and 2011) were slightly defective because of confusions between tokens in otherwise correctly executed commands.

#### Oral Expression

Oral expression was assessed by means of the French version of the BDAE (Mazaux and Orgogozo, 1983) and the Bachy 36-items naming test (Bachy-Langedock, 1989). In July 2010, performance on most oral language tasks was severely hampered by a complex speech disorder combining (a) a stutter-like behavior with articulatory efforts in initiating words, associated with spectacular facial synkinesias, (b) an impressive grammatical disorder in spontaneous speech and across all tests administered (including oral repetition and reading aloud tasks) that was observed in L1, but not in L2 and L3, and (c) a foreign accent, which was perceived by the neurolinguist as either English or Slavic, and which similarly only affected her native language. Of note, the patient did not produce one single paraphasia in the sentences generated during the entire oral language assessment, and obtained normal results on automatized sequences (counting days of the week and months of the year), a responsive naming task (word finding upon orally presented questions), a body-part naming task, and a semantic verbal fluency task (1 and 2 min generation of animal names).

Overall performance in oral language was roughly similar in May 2011, though the foreign accent, articulatory efforts, and stutter-like behaviors had considerably diminished at that time. However, a prominent and paradoxically fluently produced grammatical disorder was still noticed in spontaneous speech and during all language tasks. Again, paraphasic errors were not observed.

In August 2012, 1 month after an appendectomy under general anesthesia, the patient was referred to the neuropsychological department by her neurologist, who was astonished by the unexpected and unexplained improvement of her oral language skills. The grammatical disorder in spontaneous speech and oral language tasks had completely disappeared, as had the foreign accent. Sporadically, a discrete and short-lasting stuttering was observed. Results on oral language tasks were well within normal limits, except for a persistently weak performance on visual confrontation naming and a decreased generation of animal names (paradoxical reduction of semantic verbal fluency in association with a spectacular improvement of oral expression) (Table 4).

#### Reading

Reading aloud in 2010 and 2011 (assessed by means of the BDAE) was effortful mostly because of the stutter-like symptoms and was characterized by a foreign accent. Moreover, reading sentences was contaminated by massive grammatical errors. The words composing the sentences, however, were correctly read. As was the case in spontaneous speech, in 2012 reading aloud had completely normalized. Reading comprehension of sentences and paragraphs was normal in 2010 and 2011. Unexpectedly, the patient performed worse at the time oral language and reading aloud had normalized (Table 4).

#### Writing

In written language production (assessed by means of the BDAE), graphomotor skills and writing words upon dictation were normal at the time of the three language evaluations. Writing sentences upon dictation was altered by grammatical errors (omissions of grammatical words and use of infinitive verbs), but the words themselves were written flawlessly. The written description of the Cookie Theft picture remained grammatically impaired over time, though, again, all individual words were spelled correctly.

### Phonetic Assessment

The first author (Stefanie Keulen) performed a perceptual analysis of 5 min of spontaneous speech during which the patient explained her medical history, in order to seek which segmental and suprasegmental features could have induced or at least reinforced the impression of accent foreignness. To this purpose, the excerpt was transcribed into International Phonetic Alphabet. As the patient’s foreign accent was judged to have diminished as of 2011, a sample was selected from the recordings made in 2010.

Perceptually, the patient appeared to realize the French uvular /R/ as an English diphthong. For instance, the verb *faire* (/fεR/) (to do) was pronounced as /feә^r^/. On other occasions, she used excessive alveolar trill (as, for instance, in Italian, Spanish, or Russian) instead of uvular rhoticity. Other segmental errors consisted of additions of [r] (devoir → dev*r*oir) and schwa (plus → p*e*lus) (epenthesis). The patient sometimes used a voiced velar fricative (/γ/) instead of the voiced velar plosive /g/ (e.g., /γrɑm/ for /*g*Rɑm/ or “gram” in English), which could have induced the impression of a Dutch/Flemish-like accent. Moreover, she produced voiceless and voiced ejective consonants as, for instance, in /k’ɒm/ (*comme*; like), /bRεIk’dæns/ (*breakdance*), and /beg’εje/ (*bégayer*; to stutter). Ejectives are highly uncommon in European languages and occur in some languages in the region of the Caucasus and the Americas (Hayward, 2013). The patient equally spoke with a strangled voice, probably reinforced by the repeatedly produced egressive, glottalic airflow which caused the realization of the ejectives, instead of the typical, expected pulmonic egressive airstream. Intonation of speech was aberrant. Word accent was sometimes wrongfully placed (e.g., *beau*coup; many). Melody of speech was equally altered in 2010, and there were sudden excursions of speech intensity.

## Experiment

### Aim

A perceptual accent rating and attribution experiment was set up with the purpose of disclosing (a) whether a group of French-speaking listeners judged the patient to speak with a foreign accent, (b) which accents could possibly be identified in the FAS speaker’s speech, and additionally (c) how native and non-native speakers of French could be identified. Because of the severe speech impediment suffered by the patient, we decided to apply Dankovičová and Hunt’s (2011) procedure to select the stimuli (*see below*).

### Methods

#### Materials and Samples

This study consisted of a perceptual experiment in which 25 French-speaking students in French linguistics at a francophone university in Brussels – who were not formally acquainted with speech pathologies of any kind – blindly assessed the (foreign) accent and linguistic background of six speakers. One speaker was the FAS patient, whose stimuli were mixed with stimuli from five other speakers: one was a native French-speaking Belgian woman stemming from the same geographic area as the patient, and four others were non-native speakers of French with an audible foreign accent.

The selected stimuli were retrieved from a recorded informal interview, which took place in 2010 in the context of neurolinguistic testing. The patient explained her medical history, symptoms, and the chronology of events. Nine isolated words and six grammatically correct utterances were selected and edited as to ensure full anonymity (Dankovičová and Hunt, 2011). Only correct utterances were chosen in order to avoid any possible artifacts in the listeners’ judgments. In total, 90 stimuli were presented to the raters (15 stimuli × 6 speakers). Files were adjusted for the purpose of assessment using PRAAT, version 5.4 (PRAAT for Mac; Boersma and Weenink, 2014).

#### Control Speakers

Five female control speakers (Table 5) read the words and utterances selected from the patient’s interview. Recordings were made with a Marantz Professional PMD 661 portable recorder and adjusted *via* PRAAT (Boersma and Weenink, 2014). The non-native speakers of French were, respectively, of Belgian (Dutch), English, German, and Chinese origin. In accordance with Verhoeven et al.’s (2013) methodology, their foreign accents had not been matched to those the medical staff had tentatively reported in the patient. It was assumed that most listeners would be acquainted with the control speakers’ accents.

**Table 5:** **Demographic data of speakers (FAS and controls) in the perceptual accent rating experiment, including an indication of the level of French, CEFR, Common European Framework of Reference for Languages (Council of Europe, 2001)**.

Nature	Gender	Age	Country of birth	Mother tongue	Level in French (CEFR)
FAS	F	40	Belgium	French	–
Control 1	F	37	Belgium	French	–
Control 2	F	48	Belgium	Dutch	B1+
Control 3	F	52	Germany	German	B1+
Control 4	F	48	China	Mandarin Chinese	A2+/B1
Control 5	F	42	England	RP English	A2+/B1

#### Stimuli and Assessment

Total sample time was 25 min. and 26 sec. The stimuli were separated from one another by a 15-s interval to allow for judgment. The sample consisted of 15 “blocks” in which each stimulus was uttered by all six speakers in pseudo-random order. Stimuli were presented only once, so each speaker recurred 15 times.

Before hearing the speech samples in open field at their institution, the listeners received the test instructions, and completed demographic information about themselves (age, gender, country of birth, time living in Belgium if not born here, mother tongue, and other spoken languages including an indication of proficiency in these languages) on a questionnaire. They were asked to rate the speakers’ degree of French-speaking “nativeness” on a seven-point scale: 1 = “definitely *not* a native speaker of French” and 7 = “definitely a native speaker of French.” In case the rating was <7, listeners were asked to identify the speaker’s mother tongue.

### Results

#### Demographic Results

Among the 25 raters (16–25 years old; mean age: 19 years and 3 months; 11 males and 14 females), 1 participant was born in England, 2 in Luxemburg, and 1 in Mali. However, they all were raised and educated in French, except for the English student (aged 17), who was raised bilingually (French–English) but had been living in the French-speaking part of Belgium for 16 years.

#### Accent Rating Results

Results were loaded into SPSS version 22 for Mac OS X (Corp, 2013). First, inter-rater reliability was calculated for each speaker. As we had 25 different raters, this was examined by means of an intraclass correlation coefficient (ICC). As each item was assessed by each rater, and raters were randomly selected (sample selection, not population), the two-way random model was applied, checking for agreement implying that systematic differences between raters were taken into account. Results demonstrated that for FAS ICC (2,25) = 0.77, for French ICC (2,25) = 0.798, for Dutch ICC (2,25) = 0.948, for German ICC (2,25) = 0.936, for Chinese ICC (2,25) = 0.936, and for English ICC (2,25) = 0.713. These are acceptable values.

Mean scores, medians, SDs, minima, maxima, ranges, and interquartile ranges are provided in Table 6. Based on descriptive statistics, the French-speaking control appeared to be strongly associated with one extreme end of the continuum ($\bar{x}=6.653$, σ = 1.043, and *M* = 7; score 7 = “definitely a native speaker of French”), whereas the English-speaking control was clearly situated at the opposite extreme ($\bar{x}=2.056$, σ = 1.589, and *M* = 1; score 1 = “definitely *not* a native speaker of French”). The FAS patient, too, was associated more often with an elevated degree of foreignness ($\bar{x}=2.288$, σ = 2.166, and *M* = 1). The remaining speakers were situated in between, they apparently were the most difficult to qualify as they were equally associated with the greatest SDs (Dutch: $\bar{x}=3.949$, σ = 2.451, and *M* = 4; German: $\bar{x}=3.880$, σ = 2.422, and *M* = 3; and Chinese: $\bar{x}=3.136$, σ = 2.164, and *M* = 3).

**Table 6:** **Perceptual accent rating experiment: mean score, median, SD, minimum (Min), maximum (Max), range, and interquartile range for the patient and each of the control speakers**.

Speaker	Mean	Median	SD	Min	Max	Range	Interquartile range
FAS	2.288	1.000	2.166	1.000	7.000	6.000	2.000
French	6.653	7.000	1.043	1.000	7.000	6.000	0.000
Dutch	3.700	4.000	2.452	1.000	7.000	6.000	6.000
German	3.880	3.000	2.422	1.000	7.000	6.000	6.000
Chinese	3.136	3.000	2.164	1.000	7.000	6.000	4.000
English	2.056	1.000	1.589	1.000	7.000	6.000	2.000

As a Kolmogorov–Smirnov test of normality showed that data were not normally distributed (for all speakers: *p* < 0.1), non-parametric statistics were applied. A Kruskal–Wallis *H* test showed that there was a statistically significant difference among ratings for the different speakers [inter-speaker difference: *H*(5) = 778.751, *p* < 0.000]. Further analysis (Mann–Whitney *U* tests) was necessary to establish inter-speaker comparisons. There was a significant difference among all speaker ratings (Table 7), except in the case of the ratings for the FAS patient (*M* = 1) versus the English-speaking control (*M* = 1): *U* = 68,166.50, *p* = 0.407, and ratings for the Dutch- (*M* = 4) versus the German-speaking controls (*M* = 4): *U* = 69,469.500, *p* = 0.771, and as such: *p* > 0.0033 (corrected *p*-value, Bonferroni correction).

**Table 7:** **Perceptual accent rating experiment: Mann–Whitney *U* scores for the individual inter-speaker comparisons**.

Group comparison	*N*	Mean rank	Sum of ranks	Mann–Whitney *U*	Wilcoxon *W*	*Z*	*p*
FAS	375	222.03	83,260.5	12,760.5	83,260.5	−21.146	0.000
French	375	528.97	198,364.5				
FAS	375	298.54	111,951.5	41,451.5	111,951.5	−10.301	0.000
Dutch	375	452.46	169,673.5				
FAS	375	300.17	112,564.5	42,064.5	112,564.5	−10.093	0.000
German	375	450.83	169,060.5				
FAS	375	324.95	121,855.5	51,355.5	121,855.5	−6.879	0.000
Chinese	375	426.05	159,769.5				
FAS	375	369.78	138,666.5	68,166.5	138,666.5	−0.829	0.407
English	375	381.22	142,958.5				
French	375	490.93	184,098	27,027	97,527	−16.233	0.000
Dutch	375	265.05	97,527				
French	375	493.14	184,928.5	26,196.5	96,696.5	−16.506	0.000
German	375	257.86	96,696.5				
French	375	525.89	197,207	13,918	84,418	−20.324	0.000
Chinese	375	225.11	84,418				
French	375	550.17	206,314.5	4810.5	75,310.5	−23.428	0.000
English	375	200.83	75,310.5				
Dutch	375	377.75	141,655.5	69,469.5	139,969.5	−0.291	0.771
German	375	373.25	139,969.5				
Dutch	375	411.27	154,225	56,900	127,400	−4.626	0.000
Chinese	375	339.73	127,400				
Dutch	375	459.42	172,281.5	38,843.5	109,343.5	−11.074	0.000
English	375	291.58	109,343.5				
German	375	408.9	153,337.5	37,787.5	128,287.5	−4.322	0.000
Chinese	375	342.1	128,287.5				
German	375	457.61	171,605.5	39,519.5	110,019.5	−10.847	0.000
English	375	293.39	110,019.5				
Chinese	375	429.47	161,053	50,072	120,572	−7.233	0.000
English	375	321.53	120,572				

**Figure 1:**
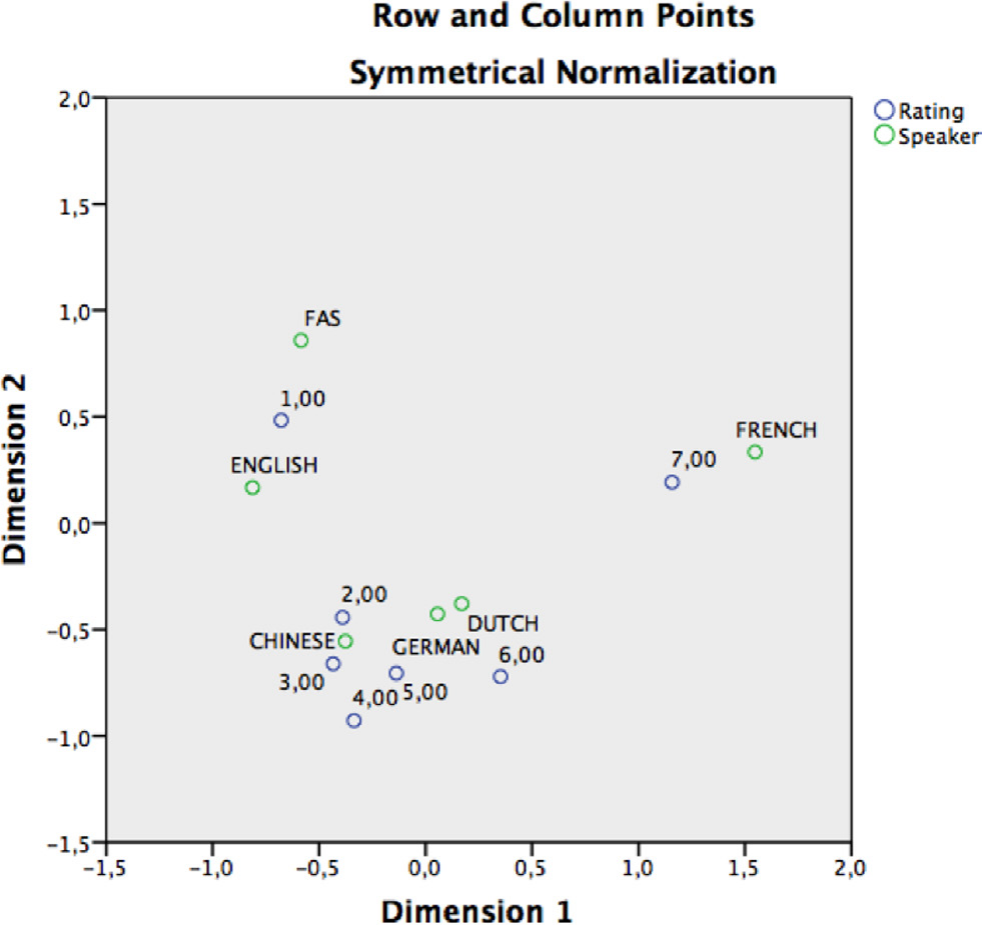
**Perceptual accent rating experiment: correspondence analysis graphically displaying the accent dispersion and associated accent ratings in a two-dimensional space**. The points represent a vector transformation of the data displayed in Table 8. The blue circles represent the accent rating and the green circles represent the speakers. Ratings were defined as column points, speakers as row points. The distances between the scores and speakers represent the strength of association between both values. Both FAS and English are more closely associated with “Definitely non-native speakers of French” (=rating 1). French is (correctly) associated with “definitely a native speaker of French” (=rating 7).

A correspondence analysis^6^ (Clausen, 1998) in which the FAS patient and the control speakers represent the first categorical variable, and the ratings attributed to them (7 = “definitely a native speaker of French” and 1 = “definitely *not* a native speaker of French”) the second categorical variable confirmed that the FAS speaker and the English-speaking control were more strongly associated with accent foreignness than the other non-native speakers of French (Figure 1; Table 8). The native French-speaking control was most strongly associated with French-speaking “nativeness” (Figure 1).

**Table 8:** **Perceptual accent rating experiment: correspondence table presenting the frequency of each response (1, 2, 3, 4, 5, 6, or 7) for the patient and each of the control speakers**.

Correspondence table							

Rating	Speaker						

	FAS	French	Dutch	German	Chinese	English	Active margin
1	246	4	97	100	136	217	800
2	30	5	52	46	47	53	233
3	21	5	37	43	52	45	203
4	10	2	24	24	31	15	106
5	11	15	28	39	36	28	157
6	5	25	26	15	25	6	102
7	52	319	111	108	48	11	649
Active margin	375	375	375	375	375	375	2250

*The data are transformed to vectors in a two-dimensional space (Figure 1)*.

#### Accent Attribution Results

**Figure 2:**
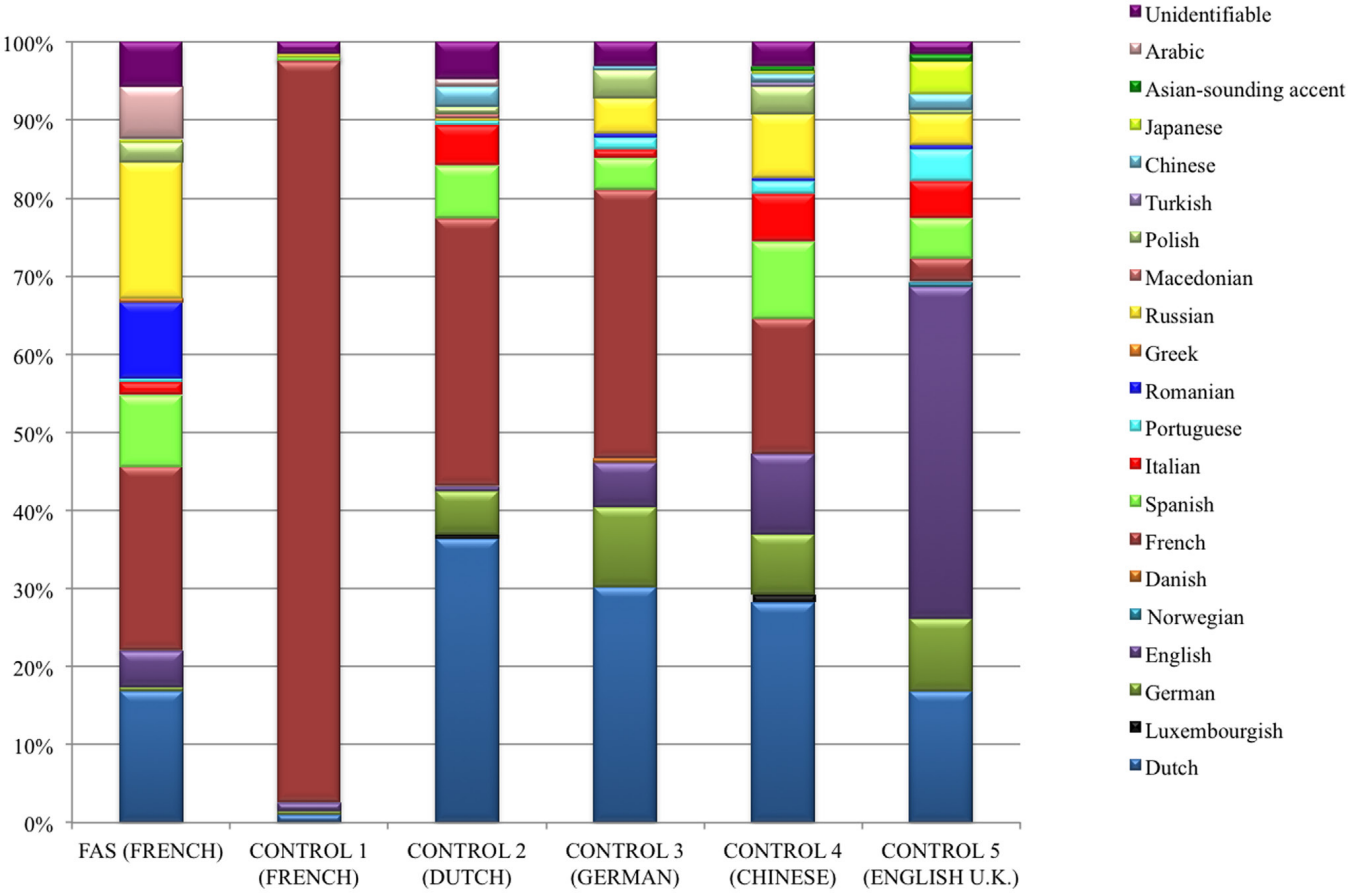
**Graphic representation of the stratification of the different native languages which 13 native French-speaking raters associated with the stimuli for the FAS patient and each of the control speakers**. The FAS speaker was associated with 14 different accents including French. However, comparison of the FAS patient with the native French-speaking control clearly demonstrates that the raters identified the control’s accent as their own in 95% of the stimuli versus a mere 24% for the FAS patient (see Accent Attribution Results).

13/25 (52%) raters tried to identify the origin of the accent in those control speakers they judged not to be a native speaker of French (score <7 on the rating scale) (Table 9). Figure 2 graphically displays the accent attribution of the 13 raters for all 15 stimuli per speaker (195 stimulus judgments per speaker). The native French-speaking control was recognized as a true native speaker of French in 185/195 (95%) of stimuli, whereas the FAS patient, who was also a native speaker of French, was perceived as such in only 46/195 (24%) of stimuli. In the native Dutch-speaking control, the difference between an association with a presumed French-like accent (*n* = 67/195, 34%) and a Dutch accent (*n* = 71/195, 36%) was minimal. The German-speaking control was identified as a native German speaker in only 20/195 (10%) of stimuli, whereas in 67/195 (34%) of stimuli, she was considered a French speaker and in 59/195 (30%) of stimuli, a Dutch speaker. The Chinese speaking control was identified as such in only 2/195 (1%) of stimuli. She was regarded as a native speaker of French in 34/195 (17%) of stimuli, and as a native speaker of Dutch in 55/195 (28%) of stimuli. Finally, the English-speaking control was properly identified as a native English speaker in 83/195 (43%) of stimuli, but was perceived as a Dutch speaker in 33/195 (17%) of stimuli. Speakers who were the least often associated with their native language (Dutch, German, and Chinese) were mostly given scores of 3 or 4 on the rating scale and had the greatest SDs.

**Table 9:** **Perceptual accent attribution experiment: number of different accent origins associated with the patient and each control speaker**.

	FAS	French	Dutch	German	Chinese	English
Dutch	33	2	71	59	55	33
Luxembourgish	0	0	1	0	2	0
German	1	1	11	20	15	18
English	9	2	1	11	20	83
Norwegian	0	0	0	0	0	1
Danish	0	0	0	1	0	0
French	46	185	67	67	34	6
Spanish	18	1	13	8	19	10
Italian	3	0	10	2	12	9
Portuguese	1	0	1	3	3	8
Romanian	19	0	0	1	1	1
Greek	1	0	0	0	0	0
Russian	34	0	1	9	16	8
Macedonian	0	0	1	0	0	0
Polish	5	0	2	7	7	1
Turkish	0	0	0	0	1	0
Chinese	0	0	5	1	2	4
Japanese	1	1	0	0	1	8
Asian-sounding	0	0	0	0	1	2
Arabic	13	0	2	0	0	0
Unidentifiable stimuli	11	3	9	6	6	3
Total (15 stimuli/speaker × 13 raters)	195	195	195	195	195	195

Assumptions about the native language of all six speakers were the least stratified in the French-speaking control. The stratification of the number of putative native accents perceived in the other speakers was fairly similar. Both the FAS patient and the Dutch-speaking control were associated with 13 different languages. The English-speaking control’s utterances were associated with 14 different languages and those of the German-speaking participant with a total of 12 different languages. Accent attribution was most stratified in the Chinese speaker, her utterances being associated with no less than 15 possible languages (see Figure 2).

A majority of the raters surmised the FAS patient’s utterances were produced by a person with a native Romance language: 87/195 (45%) of stimulus judgments were divided into 46 French, 19 Romanian, 18 Spanish, 3 Italian, and 1 Portuguese. It should be noted, however, that the Romance language family was the most familiar to the raters, who were all students in French linguistics. When accent attributions to Germanic languages (43/195, 22%) and Slavic languages (39/195, 20%) were taken together – the language families the neurolinguist associated with the FAS patient – the difference between associations with Romance languages on the one hand and Germanic or Slavic languages on the other hand appears quite small.

## Discussion

The patient we report presented with a complex set of symptoms mainly affecting her gait and verbal output, and which could reasonably not be explained by any neurologically induced deficits. The most striking speech/language symptoms consisted of a stutter-like behavior, a grammatical disorder, and a change of accent in the absence of aphasia. Interestingly, these speech/language anomalies particularly altered her native language (L1: French) and were hardly observed in L2 (Dutch) and L3 (English). Two years after the initial neurolinguistic assessment, the oral speech/language deficits unexpectedly disappeared right after the patient woke up from general anesthesia induced for an appendectomy. While acknowledging the peculiar interest of the co-occurrence of accent foreignness and grammatical anomalies in psychologically induced speech-output disorders (Van Borsel et al., 2005; Verhoeven et al., 2005; Poulin et al., 2007), in the present study, we purposely focused on the patient’s change of accent^3^.

When a change of accent inducing an impression of accent foreignness originates from a pathological condition, it is called FAS. FAS is “a motor speech disorder in which patients develop a speech accent which is notably different from their premorbid habitual accent” (Verhoeven and Mariën, 2010; p. 600). Verhoeven and Mariën (2010) classified FAS into three distinct taxonomical types: neurogenic, psychogenic, and mixed. In *neurogenic FAS*, the change of accent is associated with damage to the central nervous system. As such, it corresponds to the prototypical FAS as defined by Whitaker (1982). In *psychogenic FAS*, there is no evidence of neurological damage, and the accent change is ingrained in underlying psychological issues or psychiatric disorders. In *mixed FAS*, a neurologically induced accent change brings about psychological adjustments aiming at improving the authenticity of the newly acquired accent in order to create a more coherent new personality. This taxonomic differentiation has important implications for the management of treatment strategies.

In the current study, the patient’s accent foreignness was affirmed by 25 independent, native speakers of French on the basis of an accent rating and attribution experiment. A majority of stimuli (76%) spoken by the FAS patient were assigned a non-French accent (whereas 95% of stimuli spoken by the French-speaking control were allocated a French accent). The stratification of the number of putative accents perceived in the FAS patient and the non-French-speaking controls was fairly similar (Figure 2). This finding also demonstrates the patient’s strong accent foreignness, and corroborates the results of the phonetic assessment, which identified several segmental and suprasegmental transformations affecting the patient’s speech output.

We consider the patient reported in the present study to represent an instance of psychogenic FAS for several, not mutually exclusive reasons:

1. The sudden and unexpected remission of all oral verbal output anomalies immediately after waking up from general anesthesia seems hard to explain on neurological grounds. Although the impact of general anesthesia on cognitive functions is still a matter of opinion (Guay, 2011), one would expect such an impact, if any, to induce (transitory) post-operative cognitive defects rather than improvements (Monk et al., 2008).
2. The selective, post-operative normalization of oral expression compared to the persistent grammatical disorder in written expression remains puzzling, especially in the absence of aphasia. Typically, spoken and written productions display similar error patterns in agrammatic aphasia subjects (Goodglass, 1993; Turkstra and Thompson, 2011). To the best of our knowledge, the exceptional instances in which a production deficit selectively spared grammatical utterances in an output modality, while selectively affecting them in the other, have only been reported in the context of aphasia (Miceli et al., 1983; Goodglass, 1993; Rapp and Caramazza, 1997; Miceli, 1999; Vandenborre and Mariën, 2014).
3. In the absence of aphasia, the presence of an accent change, a grammatical disorder, and a stutter-like behavior selectively disrupting the patient’s mother tongue, while preserving L2 and L3 is also remarkable. Linguistic deficits disturbing one language more than the other(s) in multilingual speakers are a well-known phenomenon in the context of bilingual or polyglot aphasia (Paradis, 1995; Fabbro, 2001, 2002; Leemann et al., 2007; Gatignol et al., 2009). Up to the present, however, it remains speculative whether such selective speech-output defects are also to be expected in non-aphasic, brain-lesioned patients.
4. Although the analysis of the patient’s grammatical disturbances falls outside the scope of the current study, the bizarre and inconsonant pattern of her speech errors might shed an additional light on the nature of her verbal output disorder. As was the case in Cottingham and Boone’s (2010) patient, the current patient also inconsistently split numbers into separate digits. For instance, she admitted having been addicted to psychoactive substances for 12 years (pronounced “douze”; *twelve*), which caused her to lose weight and to weigh 39 kg (pronounced “trois neuf” [*three nine*] instead of “trente-neuf” [*thirty-nine*]). When asked in what year she had been admitted to one of the psychiatric institutions, she answered “en deux zéro zéro un” (*in two zero zero one*) instead of “en deux mille un” (*in two thousand and one*).
5. The patient’s mood apparently also influenced the characteristics of her spoken utterances, as she was able to shout grammatically correct sentences without foreign accent or stuttering during a violent fit of anger. Short utterances of normally produced spoken language can be observed in aphasic patients displaying automatic-voluntary dissociations between automatic and propositional language use (Basso, 2003). However, the patient’s outcries in this context clearly do not match short and automated or highly learned utterances.
6. Lesion-induced language and communication impairments are known to be at risk of occasioning emotional, behavioral, and psychosocial problems (Carota et al., 2002). Miller et al. (2011) showed that in neurogenic FAS, the accent change impacted on the patients’ daily functioning by generating feelings of loneliness, depression, frustration, and loss of confidence. In spite of the nature and severity of her expressive difficulties, however, the current patient never showed frustration or dislike in relation to her verbal output impairment. She enjoyed the attention her speech disorder received and always willingly participated in the neurolinguistic assessments. She did not try to avoid social contacts.
7. Repeat neurological examinations could not demonstrate any neurological deficits, and structural brain imaging studies repeatedly failed to disclose supratentorial and infratentorial lesions. Unfortunately, we could not obtain functional brain imaging data in the patient. In the event of a metabolic dysfunction or other subtle lesions not detected by structural brain imaging, such investigations might possibly have revealed functional abnormalities (e.g., brain perfusion or metabolism defects) in morphologically undamaged regions of the motor speech production circuitry at the time the verbal output disorder was present (Moreno-Torres et al., 2013). However, we find it difficult to relate the putative occurrence of such unexplored functional abnormalities in the distributed anatomical network controlling motor speech production to the selective impairment of phonetic/phonological components perturbing only one of the three languages spoken by the patient. Alario et al. (2010) admittedly showed that cognitive-based syllabic representations are separate in early bilinguals but are shared across languages in late bilinguals. Early bilinguals would have independent cognitive syllable representations allowing them to approach separate monolingual phonetic patterns in L1 and L2 (this might possibly theoretically explain the foreign-accent selectiveness in our patient on cognitive grounds), and late bilinguals would use the same cognitive representations when speaking either language. Alario et al. (2010) assumed that cognitive syllable representation would originate from the bilingual speaker’s earlier L1 experience, which would be appropriate for L1 but only approximate for L2. However, according to this hypothesis, if one accepts the possibility of foreign-accent selectiveness in L1, one would expect a similar accent foreignness in our patient’s L1 and L3 as she was a late learner of English. We could not observe the latter accent pattern in our patient.
8. A possible lesion-induced disruption of neurotransmitter activity and a potential influence of pharmacological interventions have been hypothesized to play an imaginable role in the appearance and resolution of FAS symptoms (Moreno-Torres et al., 2013). These assumptions are grounded in the observations that on the one hand, increasing cholinergic activity has been shown to facilitate recovery from post-stroke aphasia and apraxia of speech (Berthier and Pulvermüller, 2011), and that on the other hand, discontinuing neuroleptics in schizophrenic and bipolar patients has been shown to engender psychotic exacerbations with recurrence of a co-occurring FAS (Reeves et al., 2007), whereas restoring dopamine antagonists resulted in rapid suppression of symptoms (Reeves and Norton, 2001). However, the patient we report was not treated with neuroleptics during follow-up at our institution, as she never displayed psychotic signs since she was seen for the first time in 2010. Two months before the appendectomy performed in a peripheral hospital, a treatment with amisulpride was initiated by her psychiatrist because of the emergence of a depressive mood. Yet, there appeared to be no pharmacological effect on the speech-output disorder, as this did not change characteristics, and spontaneously and dramatically resolved only 2 months later, right after the surgical intervention.

Given the above-listed arguments, we strongly believe the accent foreignness in the reported patient to be of psychogenic origin. Although a conversion disorder could not formally be confirmed by means of an MMPI, repeated neurological and psychiatric observations, and follow-ups all clearly pointed to psychogenic behavioral and speech/language disturbances.

## Conclusion

In 1982, Whitaker proposed four criteria which a patient should meet in order to be diagnosed with FAS (Table 1). In the current paper, we report on a non-aphasic patient with FAS who only partly satisfied these criteria. The patient’s accent was – in accordance with Whitaker’s first criterion – perceived as “foreign” by medical staff, friends, and relatives, as well as by a group of 25 independent, native French-speaking listeners who rated her accent in a perceptual rating and attribution experiment. However, the two following criteria were challenged, in that we could not find any evidence of a *clear* cerebral insult to explain the sudden arousal of the accent. In addition, the patient was an unbalanced polyglot speaker of three languages, which is defying Whitaker’s fourth criterion. As regards this last criterion, in the current experiment, the patient’s accent was associated with no less than 13 different languages, indicating that in polyglot FAS patients, listeners do not necessarily attribute the provenance of the perceived accent to one of the languages spoken by the patient. In fact, identifying the origin of the perceived foreign accent in FAS patients appears to depend on the degree of exposure of listeners to foreign accents (Di Dio et al., 2006; Miller et al., 2006; Verhoeven et al., 2013).

In the present study, it is also remarkable that the accent foreignness, along with the grammatical disorder and the stutter-like behaviors particularly affected the patient’s mother tongue (French), whereas these anomalies were hardly observed in L2 (Dutch), and L3 (English). Furthermore, the occasional loss of foreign accent (and other speech anomalies) when the patient was emotionally distressed was quite noteworthy. In addition, the sudden and unexpected resolution of the foreign accent after the surgical intervention remained quite puzzling, as was the modality-specific recovery from the oral grammatical disorder (while written expression remained grammatically altered). These and other behavioral observations in the patient all pointed to a psychogenic disorder that further contests Whitaker’s third criterion. The latter was also called in question by at least five reports of FAS in association with a conversion disorder published between 2005 and 2011 (Verhoeven et al., 2005; Tsuruga et al., 2008; Cottingham and Boone, 2010; Haley et al., 2010; Jones et al., 2011).

As we conclusively demonstrated, the patient reported here suffered from a speech-output disorder which listeners perceived as foreign-accented. The origin of the patient’s accent foreignness and her multilingualism led us to conclude that Whitaker’s operational definition of what he called “foreign accent syndrome” (Whitaker, 1982; p. 195) is too restrictive and outdated. Whitaker’s criteria appear not to offer enough space to include the currently accepted taxonomic variants of FAS (Verhoeven and Mariën, 2010). Even for the neurogenic subtype, the last criterion seems barely maintainable, as polyglot, brain-injured FAS patients have also been reported (Schiff et al., 1983; Avila et al., 2004; Paquier and Assal, 2007; Levy et al., 2011). These findings underscore the necessity for a solid clinical diagnosis in the light of further treatment.

## Author Contributions

Conception and design: SK and PP. Acquisition of data: SK, NM, and PP. Analysis and interpretation of data: SK, JV, RJ, and PP. Drafting the manuscript: SK and PP. Critical manuscript revision: all authors. Critical revision of reviewed manuscript: SK and PP. Final manuscript approval: SK and PP on behalf of all authors.

## Conflict of Interest Statement

The authors declare that the research was conducted in the absence of any commercial or financial relationships that could be construed as a potential conflict of interest.

## Footnotes

^1^The American Speech-Language-Hearing Association (ASHA) defines fluency as “the aspect of speech production that refers to the continuity, smoothness, rate, and/or effort with which phonologic, lexical, morphologic, and/or syntactic language units are spoken” (American Speech-Language-Hearing Association, 1999).

^2^For instance, the number 11 was uttered as “one-one.”

^3^Though the grammatical disorder is a noteworthy aspect of the patient’s language profile, the analysis of its multimodal characteristics is beyond the scope of the current study and will be reported elsewhere.

^4^Regulations and procedures to be found at http://www.erasme.ulb.ac.be/page.asp?id=9536&langue=FR.

^5^These speech problems showed up when the patient was refused additional financial support by an official social insurance company.

^6^Correspondence analysis is a technique, which evaluates “the association between two or more categorical variables by representing the categories of the variables as points in a low-dimensional space. Categories with similar distributions are represented as points that are close in the space, and categories that have very dissimilar distributions are positioned far apart” (Clausen, 1998, p. 2).
